# Perceptions of the Role of Short-Term Volunteerism in International Development: Views from Volunteers, Local Hosts, and Community Members

**DOI:** 10.1155/2016/2569732

**Published:** 2016-06-12

**Authors:** Bethina Loiseau, Rebekah Sibbald, Salem A. Raman, Benedict Darren, Lawrence C. Loh, Helen Dimaras

**Affiliations:** ^1^Human Biology Program, Faculty of Arts & Science, University of Toronto, Toronto, ON, Canada M5S 3J6; ^2^Division of Clinical Public Health, Dalla Lana School of Public Health, University of Toronto, Toronto, ON, Canada M5S 1A1; ^3^The 53rd Week Ltd., Brooklyn, NY 11249, USA; ^4^Department of Ophthalmology & Vision Sciences, Faculty of Medicine, University of Toronto, Toronto, ON, Canada M5T 3A9; ^5^Department of Ophthalmology & Vision Sciences, The Hospital for Sick Children, Toronto, ON, Canada M5G 1X8; ^6^Department of Human Pathology, College of Health Sciences, University of Nairobi, Nairobi 00200, Kenya

## Abstract

*Background.* Short-term international volunteer trips traditionally involve volunteers from high-income countries travelling to low- and middle-income countries to assist in service-related development activities. Their duration typically ranges from 7 to 90 days. The city of La Romana, Dominican Republic, receives hundreds of short-term international volunteers annually. They participate in activities aimed at improving conditions faced by a marginalized ethnic-Haitian community living in* bateyes. Methods.* This qualitative analysis examined perceptions of short-term international volunteerism, held by three key stakeholder groups in La Romana: local hosts, international volunteers, and community members. Responses from semistructured interviews were recorded and analysed by thematic analysis.* Results.* Themes from the 3 groups were broadly categorized into general perceptions of short-term volunteerism and proposed best practices. These were further subdivided into perceptions of value, harms, and motivations associated with volunteer teams for the former and best practices around volunteer composition and selection, partnership, and skill sets and predeparture training for the latter.* Conclusion.* Notable challenges were associated with short-term volunteering, including an overemphasis on the material benefits from volunteer groups expressed by community member respondents; misalignment of the desired and actual skill sets of volunteers; duplicate and uncoordinated volunteer efforts; and the perpetuation of stereotypes suggesting that international volunteers possess superior knowledge or skills. Addressing these challenges is critical to optimizing the conduct of short-term volunteerism.

## 1. Introduction

Students and professionals from high-income countries are increasingly interested in working in international development [1, 2]. Growing from greater awareness of global inequities, these overseas experiences come in many forms, varying in duration and objectives. One controversial manifestation is short-term international volunteering, in which individuals of varying skill and training from high-income countries (HICs) volunteer over 1 to 12 weeks in a low- or middle-income country (LMIC), commonly with a service-oriented agenda [3, 4].

Sometimes called “voluntourism,” short-term international work is often criticized for many reasons. Broader sociopolitical critiques suggest that short-term volunteering trips might reinforce postcolonialist relationships between the Global North and the Global South [5–7], demonstrated by a unidirectional flow of volunteers and material provisions from North to South. Practical concerns surround the conduct of such trips, suggesting that they may encourage volunteers to engage in unethical practices, mismatch unskilled volunteer efforts to skilled labour needs, and foster host community dependence on volunteers contributions, all while providing limited actual benefit to the communities themselves [8–11]. Assessing these criticisms is hampered by an absence of research and evaluative frameworks that effectively assess the magnitude and persistence of outcomes achieved by short-term volunteering trips.

A popular community that receives voluntourism groups is the ethnic minority population of Haitians in the city of La Romana, Dominican Republic. Largely employed by the sugar industry, Haitian workers and their families live in adjacent* bateyes* (residential areas) and experience significant marginalization owing to discrimination and various sociocultural, political, and geographic barriers. This has given rise to extreme community poverty and related health and development needs in the community and drawn the attention of various international volunteer groups.

Fragmented efforts and a broad focus on service without evaluation have limited objective assessments of the actual impact of these volunteer groups in La Romana. We aimed to address this gap through a qualitative inquiry that assessed various stakeholder perceptions of the purpose, outcomes, structure, and relationships that underpin these efforts.

## 2. Methods

### 2.1. Study Setting and Team

Research was conducted in La Romana, Dominican Republic, over one week in February 2014. The team included 2 faculty advisors (LCL and HD) and 4 undergraduate students (BL, RS, SAR, and BD) along with a research assistant from a local partner organization who served as cultural broker and interpreter. Research Ethics Board approval was obtained from the University of Toronto (protocol ID 28419).

### 2.2. Interview Guide

The semistructured interview guide was composed of 8 open-ended questions aimed at elucidating participant viewpoints on short-term volunteerism, including perceived value, harm, and opinions on the volunteers (Appendix). Some questions asked for feedback on how to improve short-term volunteerism.

### 2.3. Inclusion/Exclusion Criteria

Included respondents were involved in some capacity in volunteering in the local community within one of three distinct stakeholder groups:

1. *Local Hosts*: defined as persons formally affiliated with organizations in the Dominican Republic (e.g., private and/or public healthcare facilities, health-based NGOs, and orphanages) that receive foreign short-term volunteers;
2. *Volunteers*: visiting individuals from HICs participating in short-term volunteer trips in La Romana at the time of the study;
3. *Community Members*: residents of the* bateyes* and/or villages at the receiving end of the volunteer activities who were not affiliated with a receiving organization.

Individuals were excluded from participating in the study if they were under age 18 years (all groups); not formally affiliated with a locally based organization (Local Hosts); not visiting for the purpose of volunteering (Volunteers) or residents in a* batey *not served by a volunteer team (Community Members).

### 2.4. Study Participant Recruitment

Study participants were identified by purposive sampling. To recruit Local Hosts, we contacted local and visiting organizations with demonstrated involvement in short-term volunteerism. Local organizations were approached prior to arrival to coordinate meetings. Initial meetings identified key respondents, as well as additional organizations of potential relevance to contact. Recruitment of Volunteer respondents occurred either at sites related to volunteer programs or during the research team's conduct of field observations of volunteer activities in the community. Community member stakeholders were also identified during these field observations.

Informed consent was collected verbally from all participants, and a study information letter was provided. The cultural broker acted as an intermediary between the researchers and respondents and provided guidance on culturally appropriate research interactions.

### 2.5. Data Collection and Analysis

Interviews took place in various community settings and locations. Interviews with non-English speakers were facilitated by the interpreter.

Interviews conducted during the field visit were recorded in English by note-takers (BL, RS, SAR, and BD) and team debriefs after each set of interviews served to verify consistency of the collected information and to identify initial themes for further exploration during the analysis phase. The sample size for each group was capped as saturation in themes became evident.

Interview responses were digitally transcribed and the software Dedoose (http://www.dedoose.com/) was used to code relevant themes in each interview. Two authors (SAR and HD) independently coded the data. Conflicts in coding were resolved by a third, independent reviewer (BL).

## 3. Results

### 3.1. Study Participants

Thirty-three participants were interviewed. They were 18 (55%) staff members of 8 local organizations (Local Hosts), 8 (24%) laypeople from 2 communities in La Romana (Community Members), and 7 (21%) North American volunteers from 4 short-term volunteer teams (Volunteers) (Table 1).

### 3.2. Perceptions of Volunteerism and Volunteers

#### 3.2.1. Perceived Value of Short-Term Volunteerism

Themes from all three respondent categories reflected beliefs that short-term volunteer work contributed to local health and development efforts. Specific responses broadly cited examples of tangible impacts or appealed to a perceived inherent value of volunteerism.

Tangible impacts identified across all respondent categories included material support, addressing service needs and gaps, and providing specific technical expertise (Table 2). In one instance, a Community respondent identified food and medication as material benefits:

*“Teams are important to health of community because they bring good medicine. Some of the teams bring food or medication which is less expensive than at the local hospital.”*

Benefits from service or program provision were similarly identified. One Volunteer respondent broadly listed specific medical conditions targeted by their community visits:

*“Teams come to help people with diabetes, anemia, high blood pressure, and high cholesterol.”*

The provision of additional capacity or special skills to the community was another identified benefit. One Local Host respondent said:

*“[Volunteer graduate students] increase treatment and research capacity.”*

Additional service capacity for their own organizations was another benefit described by other Local Host respondents.

Expressed benefits related to the perceived inherent values of volunteer work included local empowerment and the bearing of witness to the disenfranchisement of the Haitian community. Both Community and Volunteer respondents most commonly cited “local empowerment” as a benefit, though each respondent group expressed this differently. Community respondents typically represented empowerment as increased awareness among community members of available visiting and local health services:

*“[Volunteerism] promotes [community] awareness of health facilities.”*

Volunteer respondents, by contrast, described empowerment as a potential benefit in abstract terms, without providing a practical guide to its achievement. One Volunteer respondent, in referring to empowerment, simply stated:

*“We should not make the community beggars. There is a need to empower people.”*

The opportunity to bear witness to the conditions of the* bateyes *was identified as another benefit of volunteerism by Local Host and Volunteer respondents. This benefit was typically stated without any additional elaboration on actions taken as a result of witnessing conditions, as reflected by a Local Host:

*“Volunteers act as witnesses to the conditions of the bateyes.”*

#### 3.2.2. Perceived Harms of Short-Term Volunteerism

All respondent groups could identify harms associated with short-term volunteerism (Table 2). Responses suggested that perceived potential harms might be borne both by the community (discord and dependence) and by volunteers (discouragement and safety concerns).

Regarding community discord, one Community respondent, who was also a leader in that* batey*, highlighted the potential divisions that might arise from an influx of resources from visiting volunteer groups:

*“Sometimes, people fight over the limited resources brought by the teams.”*

Alongside potential community discord, potential dependence and overreliance on volunteers was more also noted among Volunteer respondents, with one respondent stating:

*“There needs to be a fine balance between help and being a ‘provider'.”*

One Host respondent commented that short-term volunteer work might result in discouraging volunteers, particularly those that visit regularly:

*“Some work done by prior teams is ‘undone' during continual developments, and when those team come down, they cannot see the fruits of their labour, which can be disheartening to that team.”*

Volunteer respondents identified potential harms to themselves directly from certain community elements. They believed that they could indirectly cause the community harm, if the harm they experienced to themselves could eventually result in the discontinuation of the volunteer program. This reflected both their concern for volunteer well-being and their inherent belief that the presence of volunteers was beneficial (related to perceived benefits described earlier). As one volunteer puts it:

*“Young teenage boys in the bateyes are very flirtatious and want to have sex. Volunteers are naive, and this could lead to a dangerous situation. If dangerous things happen because of flirting, the organization could withdraw from the bateyes.” *

#### 3.2.3. Perceptions of Volunteers

In sharing their perceptions of volunteers, respondents offered opinions on volunteer motivations and the perceived quality of volunteer work. Specific to the latter, Local Host and Volunteer responses highlighted community perceptions that services offered by visiting foreigners were of higher quality than local equivalents. This was reflected in relative terms by one Local Host:

*“We [the locals] will never be same, you [the foreign volunteers] will always be higher.”*

A similar relativism was expressed by a Volunteer:

*“[Volunteers] bring North American quality [services] to the Dominican Republic.”*

Both Local Hosts and Community respondents believed that volunteers were motivated by an altruistic desire to help. A Local Host respondent described context which could easily be assumed to be motivating for the volunteers:

*“[Volunteers] see the dire needs in the bateyes. They see the children, who have nutritional issues.”*

One community respondent expressed a similar sentiment, stating:

*“They see the poverty and they want to help out.” *

### 3.3. Perceptions of Best Practices of Volunteer Work

#### 3.3.1. Team Organization

Table 3 summarizes perceived best practices identified in responses. In general, Local Host and Volunteer respondents described a number of perceived best practices for volunteer work in La Romana, such as ethical guidelines, collaboration, communication, and prior planning. Of note, Community Member respondents did not identify themes around best practices. Despite the common themes, there were notable disparities in perceptions between respondent groups. For example, though both Local Host and Volunteer respondents identified “adherence to ethical guidelines” as a perceived best practice, the actual application of this theme varied. Local Host respondents asserted a certain ethical relativism in what work could permissibly be done by volunteers, as described by one Local Host respondent:

*“[Medical interventions by untrained foreigners are] okay here. All things performed by 17 to 18 year olds are rechecked by MDs. It is shadow work. There is no ethical problem. It is an exercise in sharing and experience-building.”*

This same relativism was noted in observations from Volunteer respondents but elicited a certain discomfort and raised certain questions. One nonclinical Volunteer respondent expressed:

*“It feels a little weird [taking blood pressure measurements without formal medical training]. I assume that since it's a different country, different rules apply here. I don't think it's inherently wrong, but it is stressful. But with the right training, it gets better.”*

Other potential perceived best practices did not have the same consensus. For example, concerning duration of stay, Local Host respondents were alone in suggesting that volunteers would be more effective by staying for longer periods of time or by visiting more frequently (Table 3). Similarly, concerning guidelines around who should determine the duties of the volunteer, there was notable variance among all three groups; no one viewpoint emerged as the most common, varying from the Local Hosts, the Volunteers, the Community Members, or a combination (Table 3).

#### 3.3.2. Features of Strong International Partnerships

Another best-practice area that arose in thematic analysis was the nature of visitor-local partnerships that enabled the volunteering to occur. Respondents across all categories broadly indicated that camaraderie is critical to partnership development. Local Host respondents additionally expressed value in longer-term commitments from Volunteers. Other important features of partnership suggested by Local Host and Volunteer respondents included local leadership, equal sharing of benefits, a shared vision, and having an equal stake in the partnership (Table 3).

#### 3.3.3. Ideal Volunteer Skill Set

Another best practice related to ideal “volunteer skills” was identified by respondents. All groups commented that cultural sensitivity is an important skill for volunteers to demonstrate, as exemplified by one Community Member response:

*“[The volunteers] need to know how the people live and what they read.”*

And a similar response from a Local Host partner was as follows:

*“[Volunteers] also need to learn about the poor areas and know what kind of help is needed. They need to know what is going to be helpful.”*

While Volunteer respondents agreed that cultural sensitivity was important, some suggested a relative relevance depending on the work being undertaken; for example,

*“Cultural sensitivity is important depending on the type of volunteer team. It is essential for the medical team; sometimes for evangelical teams. It is not necessary for construction teams.”*

Local Host and Volunteer respondents highlighted the desirability of proficiency in the local language. A Local Host respondent indicated that this was reflected in the criteria applied to volunteer applicants:

*“[Admitted] volunteers must have intermediate to advanced level Spanish.” *

Though desired, it was interesting to note a spectrum of requirements for language skills. This respondent represented a Local Host organization that had the most stringent eligibility requirements for volunteers (see other—skills, Table 3). In contrast, some Volunteer respondents admitted little to no facility with local languages; one working with a different organization stated:

*“There is a language barrier; that can be worked on.”*

Interestingly, the desirability of a “willingness to help and learn” was identified only by Volunteer respondents and without any additional details as to particular technical skills that might be important (Table 3). This was contrasted by Local Host respondents which suggested that experience and technical skills were more desirable, particularly for medical and health-related projects:

*“It is better when volunteers are qualified […] as they need less local support.”*

Interestingly, despite highlighting these idealized volunteer characteristics, Local Host and Community Member respondents were often quick to point out that really anyone could help despite not meeting the ideal criteria:

*“[There is] no particular skill needed for international team.” (Local Host).*

*“The community is open to any help that the volunteer can bring.” (Community Member).*

This “anyone-can-help” mentality was best summarized by one Local Host respondent, who highlighted the careful balance between available skills, community needs, and resourcing:

*“[Volunteers] also need more education, though the people from the bateyes may give more importance to such things as food, drugs and clothing.”*

## 4. Discussion

The findings of this study provide insight into stakeholder perceptions of international partnerships and relationships that form the basis of short-term volunteering. One potential goal of the short-term volunteerism is often seen as filling gaps in local services in the short-term, while building sustainable systems to promote self-sufficient communities in the long term. In that vein, respondents universally agreed that “volunteering is good,” offering a variety of reasons to support this concept (Table 2). However when asked* why* volunteering was good, responses focused largely on material resources that the volunteers bring to the community and less on sustainability. It seems problematic that this was overwhelmingly the primary benefit noted by respondents irrespective of which group they were categorized in (Table 4), suggesting that the underlying belief is that the donations that arrive with volunteers are more plentiful than what might be sent without them, if any at all. Related to this, other perceived benefits were not cited as consistently as the idea of increased material support, and, specifically, these other benefits were most often identified by Local Host or Volunteer respondents, who arguably are more invested in justifying the practice of volunteerism than the Community Members.

Limited feedback from Community Member respondents might lead one to question whether Community Members are true partners in the conduct of short-term volunteering (Table 4). While Local Hosts and Volunteer responses heavily tied to themes around mutual benefits, strong partnerships, equal stakes, and benefits in the partnerships, local community members were unable or chose not to offer detailed perceptions on what makes a good partnership. This suggests that they may not be true partners, such that their voice is heard and they retain a sense of ownership and control in the relationship. Instead, it is possible that the Local Hosts and Volunteers, working in partnership, organize themselves and “act upon” the local community.

This poses challenges to increasing the involvement of the local community in directing this phenomenon; visiting volunteers may already feel like they are already in partnership with the community by perceiving that the local organization is synonymous by proxy for the community's voice. The absence of the local community in the partnership may be because only the organization and volunteers have invested time and energy into developing, designing, and executing the volunteer programs. While these aim to help the community, a failure to engage might lead community members to view themselves as passive recipients, in turn limiting their willingness to assume ownership, participate actively, and foster greater self-empowerment [12]. This is supported by Social Identity Theory, which suggests that interactions between advantaged and disadvantaged populations can negatively influence the ability of the disadvantaged population to act collectively to improve their own conditions [13] owing to the disadvantaged group believing that it is not possible to become a member of the advantaged group. While we did not gather direct evidence in support of this in La Romana, it may explain why community respondents' mentioned only material benefits of short-term volunteerism and did not suggest benefits of improved social standing or capacity, like the Local Hosts and Volunteers did. In addition, organizers and volunteers commented about “bearing witness” to the conditions in the* bateyes*, demonstrating that they held a “romantic notion” of their overall impact on the community. Meanwhile, they did not recognize the potential opposite effects their presence might have had on the community.

The Local Hosts and Community Members cited the need for more teams and more resources. Their comments, however, indirectly pointed to the potential for creating an unsustainable overreliance and uncoordinated effort or even harm to the local community (Table 4). Ensuring that volunteer activities are evaluated by measurable outcomes and tangible impact will support best global health and development practice that sees solid partnerships as crucial to success [14].

It was interesting that both Local Hosts and Community Members suggested that “anyone can help” in the context of volunteerism. When interpreted in the context of the overarching “benefit” of volunteerism (i.e., increased material resources) it follows that a specific set of volunteer skills was not considered to be essential. Volunteers, however, tended to think more about what they personally could offer and “learn” to offer; this was not something that the Local Hosts or the Community Members identified. The lack of general consensus on what makes a suitable volunteer suggests unclear goals and direction (Table 4).

Then there is the issue of improving quality of services in the* bateyes*, the perception that appears to keep volunteers returning to help. One volunteer stated that international volunteers bring North American quality services to the local population; a Local Host participant even went so far as to say that foreign volunteers are “superior” to their local counterparts. This perception almost certainly drives the phenomenon of unskilled student volunteers engaging in medical procedures for which they were not formally trained, which falsely confers superiority as an “expert” on these volunteers (Table 4), regardless of their actual qualifications [15].

The problems identified here may cause volunteers to observe development issues only through a lens of Western privilege, which aligns with the literature suggesting that the conceptualization of development has been vastly simplified by agencies that send volunteers to the Global South and upheld by the receiving host organizations [16–18]. Development is often marketed as “making a difference” wherein specific skills are not really required, but a desire to help is [16]. This thereby justifies the volunteer work done by unskilled individuals (mostly youth) within international settings as a development “solution” [16]. When international volunteers participate in these types of projects and fail to self-reflect or question the actual impact that their services and/or presence have on the community, these elementary notions of development are perpetuated. Evidence of this was observed in our study when none of the volunteers could identify any potential harms that international volunteering might have to the local community; only indirect harms associated with community members not having access to volunteer services for whatever reason (Table 2). In fact, the only harms that were named were those to the volunteers themselves (Table 2).

## 5. Conclusions

This study has identified many challenges facing short-term volunteering in La Romana, which have application to similar phenomenon in other communities. These challenges suggest that current paradigms of short-term international volunteerism would benefit from change. To that end, the application of a Theory of Change Framework, a methodology used to plan for and promote social change [19, 20], may provide guidance around addressing concerns to optimize the impact of volunteer efforts on the target community. In La Romana, we observed continued interest in short-term volunteerism and an obvious need for improvement of conditions in the* bateyes*. Whether or not the former can help achieve the latter remains to be seen; further research and the application of a Theory of Change could help assess dynamics between the Local Host, Community Members and international Volunteers and identify ways to construct short-term volunteer efforts that contribute to an identified goal with measurable, lasting outcomes.

## Figures and Tables

**Table 1:** Study participants' demographics.

Category	Study participants	
	*n*	%
Local organization	18	55%
*Organization 1*	*4*	*22%*
*Organization 2*	*4*	*22%*
*Organization 3 *	*1*	*6%*
*Organization 4*	*3*	*17%*
*Organization 5*	*1*	*6%*
*Organization 6*	*1*	*6%*
*Organization 7*	*1*	*6%*
*Organization 8 *	*3*	*17%*
Local community	8	24%
*Community 1*	*4*	*50%*
*Community 2*	*4*	*50%*
Foreign volunteer	7	21%
*Team 1*	*1*	*14%*
*Team 2*	*1*	*14%*
*Team 3*	*4*	*57%*
*Team 4*	*1*	*14%*

*Total*	*33*	*100%*

**Table 2:** Perceptions of short-term volunteerism.

Themes	Local organization	Local community	Foreign volunteers
*Perceived value*			

Big “Impact” (general/vague)	“Yes [the work of volunteers] is relevant to the community. Their work brings much value.”	“They can help with everything.”	“Potential benefits are huge.”

Empower community		“[Volunteerism] promotes [community] awareness of health facilities.”	“We should not make the community beggars. There is a need to empower people.”

Provide human support (bear witness)	“Volunteers act as witnesses to the conditions of the bateyes.”		“When volunteers come, they spread the word to the people they know and the foreign teams that come will multiply in number.”

Provide specific expertise	“Medical volunteers are highly sought after. There is a dire need for medical help and expertise from OB/GYN, dentists, psychologists.”	“Medical teams can provide care at lower cost.”	“Teams come to help people with diabetes, anemia, high blood pressure, and high cholesterol.”
	“[Volunteer graduate students] increase treatment and research capacity.”		“[The local doctors] don't have the education or resources for preventative [family planning] measures so tubal ligation [service offered by the volunteer medical team] takes care of that.”

Provide material support	“The volunteer teams provide materials to the hospital.”	“Teams are important to health of community because they bring good medicine. Some of the teams bring food or medication which is less expensive than at the local hospital.”	“Partnerships increase the ‘cheerleading' effect, where more teams come down and provide more resources.”
	“The volunteer teams bring supplies.”		“Teams come with resources.”
	“They bring funding.”		“Volunteer doctors bring equipment.”
			“Volunteer teams are very necessary, especially for the bateyes. Lots of medicines are distributed.”

Expand capacity of organization	“They [volunteers] bring increased capacity to the hospital.”		
	“There is knowledge translation from foreign doctors to local doctors.”		
	“Volunteers provide education to both local students and doctors, which is very important.”		

Fills a (perceived) need/service gap	“The need is great in a poor country.”	“It's good for the community, as the community has a lot of health needs.”	“Due to the logistics that are incurred by promoting, planning and doing the surgery, [the volunteers] provide capacity [to the hospital].”
		“Teams are useful because there are poor people who don't have any opportunities to go to the hospital.”	“New construction wouldn't happen unless volunteers arrive and hire local labourers.”
		“Teams give treatment to those that can't afford health care or to those that can't afford to go to faraway hospitals.”	“Teams provide extra love to the children in the bateyes.”

Fills void left by lack of local volunteer “culture”	“It is sad that the locals do not engage in their own community. There is a need to engage the local community.”		

Benefits to volunteer	“It helps the volunteer.”		“It was a rewarding time that was beneficial to the community. I am extraordinarily grateful to have participated.”
	“Teams help enhance the students' self-worth.”		“The feeling of giving is better than the feeling of receiving.”
			“[Volunteering] brings students outside of their comfort zones and experience culture shock. It trains high school students to live in cross-cultural situations.”
			“It's an adventure, it's not a day job.”

Other	“Partnerships are beneficial to the community. Example, with American universities, this allows for building a network, or friendly partnerships, that are less formalized.”		“We help the community grow and become more stable.”
	“Volunteers are just a piece of the puzzle.”		“[Volunteer teams help to] broaden the resource network of the community.”

*Perceived harms*			

Harms to community		“Some people don't get volunteer services because they're working in the fields. They come back when it's late and the volunteers are gone.”	“Some people felt they didn't need help. I heard trash-talking from local community members who did not know I understood Creole. They said, ‘Why do we need medicines from these white people? We will get rid of those when we get home.'”
		“Sometimes people fight over the limited resources brought by the teams.”	“[Volunteerism] is becoming a bit of a ‘cottage industry'. I'm worried the pool of volunteers might dry up.”

Harms to volunteer	“Some work done by prior teams is ‘undone', during continual developments, and when those team come down, they cannot see the fruits of their labour, which can be disheartening to that team.”		“Young teenage boys in the bateyes are very flirtatious and want to have sex. Volunteers are naive, and this could lead to a dangerous situation. If dangerous things happen because of flirting, the organization could withdraw from the bateyes.”

*Perceptions of volunteers*			

Quality	“We [the locals] will never be same, you [the foreign volunteers] will always be higher.”		“The quality of international doctors is perceived as being better than the local doctors.”
	“More quality is brought by foreign medical teams. We have different experiences so we have different skill sets.”		“[Volunteers] bring North American quality [services] to the Dominican Republic.”

Motivations	“[The volunteers] see the dire needs in the bateyes. They see the children, who have nutritional issues.”	“They see the poverty and they want to help out.”	
		“They want to play [with the kids] and they want to do good.”	

Limitations of volunteers		“They usually cannot cater for workers' needs, specifically the male sugarcane workers.”	

**Table 3:** Best practices and features of international partnership.

Themes	Local organization	Local community	Foreign volunteers

*Best practices for international short-term volunteerism*			

Collaboration			“[Volunteers should work with] locals as interpreters, and should coordinate with local foundations.”

Communication	“Communication and planning is key.”		“Lack of coordination between different volunteer teams has led to unnecessary things being done and wasted resources, worried about hospital's ability to be self-sustaining.”
	“There is a need for increased coordination. There is a redundancy of efforts, for example, the same medications distributed to the same batey; this is a wasted effort.”		

Prior planning	“It is very important to have a schedule [prepared in advance].”		

Evaluation of ethical issues	“[Medical interventions by untrained foreigners are] okay here. All things performed by 17 to 18 year olds are rechecked by MDs. It is a shadow work. There is no ethical problem. It is an exercise in sharing and experience-building.”		“It feels it's a little weird [taking blood pressure measurements without formal medical training]. I assume that since it's a different country, different rules apply here. I don't think it's inherently wrong, but it is stressful. But with the right training, it gets better.”
			“I was surprised I was allowed to perform medical procedures that I would not be allowed to perform in North America. I felt it was a privilege to have this responsibility.”

Minimization of harms	“There are always safeguards so that abuses don't happen within the groups. Leaders are known by the community and there is an element of trust. There is supervision by local doctors.”		“There needs to be a fine balance between help and being a ‘provider'.”

Choice of effective volunteer duration	“Longer team [duration]s are generally better, but it depends on the type of work.”		
	“It is better if the teams stay for longer periods of time.”		
	“There is a problem with the short-term nature of the teams.”		

More frequent visits	“For the bateyes, [having] many teams [visit] is important as they bring food, medicines etc.”		
	“An increase in number of teams is desirable.”		

Determination of duties			
*Volunteer decides*	“The [organization] itself does not say which areas where help is most needed. We take the help as needed.”		“Volunteers decide the duties.”
	“Volunteers are free to decide what they feel they would like to do.”		
*Local host decides*	“For medical-related work, [the local lead doctor] decides on the duties. For bateye programs, [the local program leader] decides.”	“[To decide on volunteer duties] members of the community had a meeting and found out the health care needs.”	“The local partner decides on the duties.”
	“The [local] administration decides on the duties of the volunteers.”	“The [local] health promoter decides on the volunteer duties.”	“The local partner is probably better placed to decide on duties.”
*Both volunteer and local host decide*	“The organization's sub-director and medical director decide. We try to cater to volunteer's wishes too; in terms of area, and what they are interested to work in.”		“The local partner decides on the duties, and the Americans just help.”
	“Volunteers decide what to do but, if volunteers ask what to do, I will direct them.”		
*Other*			“God is in control. If God has called someone here to help, who am I to tell them otherwise?”

Other	“Hopefully [things will be] better in 20 years time. Better healthcare and education, so less need for international volunteers down the line.”	“Yes, I would like for teams to better cater to the community, though they always bring some form of help.”	“Flexibility of both partners is important.”

*Features of international partnership*			

Long-term commitments	“More, more, more partnerships are always good as their benefits are more long-term.”		

Local leadership			“We should work with the health promoters in the bateyes.”

Equal benefits	“Partnership has to be mutually beneficial.”		

Shared vision	“It is crucial to have such a [shared] vision especially amongst collaborating NGOs.”		“Sharing the same goals and having a passion for the mission are important.”

Equal stake	“Our partnership is a mesh network. There is real input from each partner. You can't really tease out what the specific effects [of each partner] to the overall success of the program. Everything is a piece of the puzzle.”		“We must see each other as equal partners.”

Camaraderie	“Good interaction [is a necessary component of partnership.”	“Communication and relationship must be good; need partners to work well together.”	“[Partners should be] loving and open, like a family.”
	“It is important to incorporate friendship in such connections; where there's no legal take on the relationship.”		“We need to build a relationship [with the locals].”

Other	“Need measurable results.”		“Common faith strengthens partnerships.”
			“Faith is essential to building these relationships. Religion can't be in competition with dominant beliefs of the Dominican Republic.”

*Suggested volunteer skill set*			

Willingness to help/learn			“[Volunteers need] the willingness to learn and serve in whatever capacity they can.”

Cultural sensitivity	“There is a need for a teaching component on the history and the basics of the community. For example, the change in the constitution in 2010 changed status of how a Dominican citizen is defined.”	“[The volunteers] can come with the pastor to better understand what would be the most effective help.”	“Voodoo has been noted in the bateyes; spells are casts and some houses are not visited. It is hard [for some volunteers] to understand this.”
	“Learn some history of the Dominican Republic.”	“[The volunteers] need to know how the people live and what they read.”	“Cultural sensitivity is important depending on the type of volunteer team. It is essential for the medical team; sometimes for evangelical teams. It is not necessary for construction teams.”
	“[Volunteers] should learn about poor areas in the country before coming so that [they] know what type of help to bring.”	“Volunteers need to know about community needs.”	
	“They also need to learn about the poor areas and know kind of help is needed. They need to know what is going to be helpful.”		

Language proficiency	“There are interpreters generally [to assist the volunteers], though it could be a problem without them.”		“Volunteers should know the language.”
	“Volunteers must have intermediate to advanced level Spanish.”		“There is a language barrier; that can be worked on.”
	“The ability to speak Spanish is important.”		

Technical skills	“It is better when volunteers are qualified physicians, as they need less local support.”		
	“[Volunteers] also need more education, though the people from the bateyes may give more importance to such things as food, drugs and clothing.”		

Anyone can help	“Any type of volunteer is helpful.”	“The community is open to any help that the volunteer can bring.”	
	“[There is] no particular skill needed for international team.”		

Other—skills	“General requirements for volunteers include: global health experience; intermediate or advanced level Spanish; high level of initiative, professionalism and cultural competency. Selection is made after two interviews, and review of their essay and resume.” [Note: this was for graduate or medical school volunteers of one organization.]		“One should be emotionally and spiritually ready, both before and after visiting a batey.”
			“[Volunteers] should be compliant and humble. They need to keep the bar high.”
			“[Volunteers should display] humility, and be looking to learn.”
			“Volunteers should be emotionally ready.”
			“Emotional, spiritual, physical training.”

**Table 4:** Identified problems and solutions.

Problem	Potential solution
Mismatch between aim “long-term improvement of conditions” and actual impact “short-term gain of material goods”	Clearly defined goals and carefully structured volunteer programs that try to meet those goals

Community members do not appear to be equal partners	Improved efforts to tease out the views, opinions, and needs of the community and facilitate mutual benefit

Local host and volunteers believe that more teams and resources are needed	A needs analysis to refine the problem such that appropriate long-term solutions can start to be developed; a critical examination of the motivations and conflict of interests involved

No consensus on what makes a suitable volunteer	Clearly defined goals of program matched to skilled volunteers only

Unskilled volunteers considered “superior” to locals	Critical reflection of volunteer motivations, skills, and suitability to the defined goal of the program

## Appendix

### Interview Guide

1. Do you think that foreign medical volunteers are a necessary part of health care delivery in La Romana? Why or why not? Do you think this will change in the future?
2. Can you comment on the relationship (e.g., close, distant, organized, disorganized, equal, power difference) between the foreign volunteers and the local health care workers? What about with the local community?
3. What do you think are necessary components of partnerships between people from different countries? Are there any examples in La Romana that you know of?
4. How can foreign medical volunteers best collaborate/work together with the local health care workers? With the local community?
5. How are the duties of short term medical volunteers decided upon? Do you agree or disagree with this approach?
6. Do you think that the work that volunteers do is relevant to the local community? What are some ways to ensure relevance?
7. Who chooses the volunteers who come to La Romana? How do you think volunteers should be chosen?
8. Are the results/impacts of the volunteer work shared with local health care workers? The community? Do you think this is necessary or not?
